# Relationships between the ABO blood group SNP rs505922 and breast cancer phenotypes: a genotype-phenotype correlation study

**DOI:** 10.1186/1471-2350-13-41

**Published:** 2012-05-29

**Authors:** Seth Rummel, Craig D Shriver, Rachel E Ellsworth

**Affiliations:** 1 Clinical Breast Care Project, Windber Research Institute, Windber, USA; 2 Clinical Breast Care Project, Walter Reed National Military Medical Center, Washington, DC, USA; 3 Clinical Breast Care Project, Henry M. Jackson Foundation for the Advancement of Military Medicine, Windber, USA

## Abstract

**Background:**

To date, evaluation of the association of the ABO blood group and breast cancer has yielded mixed results. SNP rs505922, located within the first intron of the ABO gene, has been associated with the adenocarcinoma subtype of pancreatic cancer. To evaluate the association between genetic variation in the ABO blood group and risk of breast cancer, rs505922 was genotyped in 629 Caucasian women with invasive breast cancer, representing a variety of clinical and pathological tumor types.

**Methods:**

Genomic DNA was isolated from blood. TaqMan SNP assay C_2253769_10 was used to determine genotypes for each patient at rs505922. Statistical analysis was performed using chi-square analysis using a P-value <0.05 to define significance.

**Results:**

Genotypes were generated for 100% of the 629 patients in this study. Allele and genotype frequencies did not vary significantly for age at diagnosis, tumor stage, size or grade, hormone, HER2 or lymph node status, intrinsic subtype, tumor type or patient outcome.

**Conclusions:**

Allele frequencies for rs505922 did not differ between women with breast cancer and published HapMap frequencies from women of European descent. Further stratification into different tumor phenotypes also failed to reveal an association between rs505922 and any clinical characteristics. Together, these data suggest that the minor allele of rs505922 and the resulting non-O blood types are not associated with increased risk or less favorable tumor characteristics or prognosis in breast cancer.

## Background

The first association between the ABO blood group and cancer risk was reported in 1953 in English patients with stomach cancer, where blood group A was associated with increased risk of stomach cancer and blood group O conferred a protective advantage [1]. A similar association between increased risk of gastric cancer and blood group A was reported as recently as 2010 [2]. In oral cancer, loss of the A and B antigens is associated with increased cell migration, in non-small cell lung cancer, the A blood group is associated with improved survival, and in ovarian cancer the B antigen is associated with increased risk [3-6].

A number of studies have investigated the role of the ABO blood group in breast cancer risk and pathology. Two early publications found no association between ABO blood group and risk of breast cancer in either English or American patient populations [6,7]. In more recent studies, the A antigen was associated with increased risk of developing invasive ductal carcinoma in 166 Greek women, while in a group of 565 Turkish women, no association was seen between breast cancer risk and any ABO blood types [8,9]. These differences in risk assessment between cancer risk and the ABO blood group may be attributed to differences in ABO frequencies between populations, the importance of using comparable case and control groups, and methods for determining ABO blood types.

In 2009, an agnostic approach to identify genes associated with risk of developing pancreatic cancer further supported the association between the ABO blood group and cancer. Genotyping of 558,542 single nucleotide polymorphisms (SNPs) in 1,896 patients with pancreatic cancer and 1,939 controls revealed a significant association between SNP rs505922, located within the first intron of the ABO glycosyltransferase (ABO) gene [10]. The protective T allele of rs505922 is in linkage disequilibrium with a single base pair deletion that encodes the O antigen, suggesting that the A and B blood antigens may be associated with higher risk of pancreatic cancer. In a follow-up study, the role of the ABO blood group was re-evaluated in 15,359 patients treated at the European Institute of Oncology with a variety of cancer types. An association with the ABO blood group was detected only for pancreatic cancer [11]. Importantly, the protective effect of the O allele was not associated with all types of pancreatic cancer but was limited to the adenocarcinoma subtype.

To determine whether that ABO blood group is associated with risk of breast cancer, SNP rs505922 was genotyped in 629 Caucasian women with invasive breast cancer diagnosed between 2001 and 2010. Data were then evaluated by a number of pathological characteristics including age at diagnosis, hormonal and HER2 status, intrinsic subtype, tumor grade, stage and size, and clinical outcome.

## Methods

### Study population

Caucasian women with invasive breast cancer diagnosed between 2001 and 2009 were selected from the Clinical Breast Care Project (CBCP) database. Blood samples were collected with approval from the Walter Reed Army Medical Center Human Use Committee and Institutional Review Board. All subjects enrolled in the CBCP voluntarily agreed to participate and gave written informed consent.

Diagnosis of every specimen was performed by a single, dedicated breast pathologist from hematoxylin and eosin (H&E) stained slides; staging was performed using guidelines defined by the AJCC *Cancer Staging Manual* sixth edition [12] and grade assigned using the Nottingham Histologic Score [13,14]. ER and PR status were determined by immunohistochemical (IHC) analysis at a clinical laboratory (MDR Global, Windber, PA) and HER2 status was assayed using the PathVysion® HER-2 DNA Probe kit (Abbott Laboratories, Abbott Park, IL) according to manufacturer’s protocols.

### SNP genotyping

Genomic DNA was isolated from blood clots using the Gentra Clotspin and Puregene DNA purification kits (Qiagen., Valencia, CA). Aliquots of DNA (10 ng) were amplified in duplicate by PCR using the TaqMan SNP assay C_2253769_10 (Applied Biosystems, Foster City, CA), representing SNP rs505922 and analyzed on a 7000 Sequence Detection System (Applied Biosystems, Foster City, CA). Genotypes (CC, CT or TT) were determined using the ABI PRISM® 7000 Sequence Detection System software (Applied Biosystems, Foster City, CA).

### Statistical analysis

Differences in genotype or allele frequencies between different clinicopathological characteristics were calculated using chi-square analysis using *rxc* Contingency Tables (http://www.physics.csbsju.edu/stats/contingency_NROW_NCOLUMN_form.html). Significance was determined using *P <* 0.05.

## Results

### Clinicopathological characteristics of the patient population

The average age at diagnosis for the 629 Caucasian women with invasive breast cancer was 58.94 years (range 28–97 years) and the majority (74%) was post-menopausal at diagnosis. Most tumors were invasive ductal carcinomas (IDCA) (70%), ER positive (78%), HER2 negative (82%) and of the luminal A (ER+/HER2-) subtype (74%). Tumors were divided between well-differentiated (33%), moderately-differentiated (38%) and poorly-differentiated (29%). Most tumors were early stage with 86% being stage I-II and 67% having negative lymph node status. Seventeen percent of the patients had died of disease.

### Frequency of rs505922 in women with invasive breast cancer

Of the 629 samples assayed in this study, six (1%) produced discordant genotypes between duplicate samples during the original run; these samples were run a second time to achieve concordance among genotypes for all duplicate samples. Allele frequencies were 0.65 for the T allele and 0.35 for the C allele. Chi-square analysis revealed that the genotype frequencies were in Hardy-Weinberg Equilibrium (*P* = 0.961). Neither genotype nor allele frequencies differed significantly from those reported for Caucasians in the CSHL-HAPMAP panel (*P* = 0.809 and *P* = 0.654, respectively).

### Evaluation of rs505922 genotype and allele frequencies with clinicopathological characteristics

Allele and genotype frequencies were compared for age at diagnosis, tumor stage, size and grade, hormone, HER2 and lymph node status, intrinsic subtype, tumor type and patient status. No significant differences were seen by genotype (Table 1) or allele (data not shown). To determine whether the minor C allele was associated with any variable, CC and CT genotypes were combined. As with genotype and allele frequencies, no significant differences were detected.

**Table 1:** Genotype frequencies by clinicopathological variable

**N = 629**	**CC**	**CT**	**TT**	**P-value**
Age				P = 0.393
<40 years	4 (12%)	11 (32%)	19 (56%)	
40-49 years	12 (9%)	59 (46%)	57 (45%)	
≥50 years	62 (13%)	211 (45%)	194 (42%)	
Tumor Stage				P = 0.143
I	49 (15%)	147 (44%)	137 (41%)	
II	18 (9%)	98 (48%)	87 (43%)	
III	11 (15%)	28 (40%)	32 (45%)	
IV	0 (0%)	5 (33%)	10 (67%)	
Tumor Size				P = 0.238
T1	56 (14%)	185 (45%)	171 (41%)	
T2	11 (8%)	66 (45%)	69 (47%)	
T3	7 (18%)	17 (45%)	14 (37%)	
Grade				P = 0.852
1	25 (13%)	83 (42%)	88 (45%)	
2	30 (13%)	104 (46%)	95 (41%)	
3	18 (10%)	77 (44%)	79 (46%)	
Hormone Status^a^				P = 0.583
ER + PR+	54 (14%)	165 (43%)	162 (43%)	
ER + PR-	10 (11%)	46 (49%)	37 (40%)	
ER-PR-	12 (10%)	57 (45%)	56 (45%)	
HER2 Status				P = 0.788
Positive	9 (10%)	38 (44%)	40 (46%)	
Negative	60 (13%)	201 (43%)	203 (44%)	
Intrinsic Subtype^b^				P = 0.748
Luminal A	54 (14%)	169 (44%)	165 (42%)	
Luminal B	4 (8%)	21 (45%)	22 (47%)	
HER2 enriched	5 (12%)	17 (43%)	18 (45%)	
Triple negative	6 (8%)	32 (42%)	38 (50%)	
Tumor Type				P = 0.196
IDCA	41 (10%)	191 (46%)	185 (44%)	
ILCA	18 (19%)	41 (44%)	35 (37%)	
Mixed ILCA/IDCA	4 (13%)	12 (40%)	14 (47%)	
Other^c^	8 (18%)	17 (39%)	19 (43%)	
Lymph Node Status^d^				P = 0.546
Negative	51 (12%)	181 (44%)	183 (44%)	
Positive	16 (11%)	74 (48%)	62 (41%)	
Patient Status				P = 0.857
Disease-free^e^	23 (13%)	85 (49%)	66 (38%)	
Recurrence	5 (13%)	19 (49%)	15 (38%)	
Dead of Disease	4 (13%)	12 (39%)	15 (48%)	

^a^Only a single patient had confirmed ER-PR + status, thus this rare tumor type was not included in the analysis.

^b^Intrinsic subtypes were defined as luminal A = ER and or PR+/HER2-; luminal B = ER and or/PR+/HER2+; HER2-enriched = ER and PR-/HER2+ and triple negative = ER, PR and HER2-.

^c^Other included histological types including tubular, medullary, apocrine, and mucinous carcinomas.

^d^Patients with isolated tumor cells (n = 33) were not included in this analysis.

^e^Only patients who have been disease-free ≥5 years were included in this analysis.

Data was not available for each variable for every patient thus “N” varies by characteristic.

## Discussion

The first suggestion of an association between ABO blood group antigens and malignancy was made almost 100 years ago, yet the role of the ABO blood group in cancer risk and prognosis remains controversial. Results from the pancreatic genome-wide association study (GWAS) in 2009 renewed interest in the ABO blood group as the T allele of SNP rs505922, located within the first intron of the ABO gene locus, was found to confer a protective advantage against pancreatic cancer. The T allele has been found to be in linkage disequilibrium with the single base pair deletion responsible for the O blood group antigen, leading the authors to suggest that people with non-O blood groups have increased risk of developing pancreatic cancer [10].

In addition to the association with blood group antigens, variability of SNPs within the ABO genomic region has been associated with circulating levels of tumor necrosis factor alpha (TNFα), soluble intracellular adhesion molecule-1 (sICAM-1) and alkaline phosphatase [15-17]. Because these tumor-promoting factors may also provide an environment favoring breast tumor development, variation of rs505922 may contribute to increased risk of breast cancer development. Comparison of allele frequencies in our cohort of 629 Caucasian women with invasive breast cancer, however, did not differ significantly from Caucasians in the HapMap project, suggesting that the minor allele of rs505922 is not associated with increased risk of breast malignancy.

In the follow-up paper by Iodice et al., which found no association between ABO blood group and breast cancer risk (P = 0.60) in 7,208 women treated at the European Institute of Oncology (IEO), the importance of tumor heterogeneity was highlighted [11]. While the GWAS found an association between rs505922 and pancreatic cancer overall, evaluation of ABO blood group antigens with subtypes of pancreatic cancer in the IEO revealed that non-O blood types were associated with the exocrine, but not the endocrine, form of pancreatic cancer. Breast cancer is marked by tremendous heterogeneity in terms of age at diagnosis, tumor pathology, and molecular characteristics. Thus, the ABO blood group may be associated with increased risk or prognosis of certain subtypes of breast cancer. Neither allele nor genotype frequencies differed significantly by age at diagnosis, tumor stage, grade and size, hormone, HER2 and lymph node status, intrinsic subtype or prognosis suggesting that the ABO blood group is not associated with either overall risk of or certain subtypes of breast cancer. It should be noted, however, stratification of genotyping data into phenotypic groups did, for certain pathological characteristics, result in small sample size (e.g., when stratified by subtype, only 4, 5 and 6 patients with the CC genotype had luminal B, HER2-enriched and triple negative tumors, respectively). Detection of association between genotype and phenotype where the sample size was small could, therefore, be masked.

Our results are in agreement with those from Iodice et al. derived largely from an Italian patient population as well as those from Dede et al. who found no association in 565 Turkish women [8,11] In contrast, Stamatakos et al. found an association between the A antigen and IDCA and/or poor prognosis in population of Greek women [9]. Differences between the Greek study and the other studies that failed to find an association may reflect the small sample size used in the Greek study, which was based on 166 women with breast cancer, which is much smaller than the 565 Turkish, 629 Caucasian American, or 7,208 Italian women included in the other studies.

Although this study does not support a role for the ABO gene in breast cancer incidence or prognosis, this study was limited to individuals with self-reported “white” ancestry. The frequency of the ABO blood group varies globally, thus, differences in allele frequency of the ABO blood group must be considered when evaluating the role of ABO in disease. For example, the A antigen, which was found to be associated with increased breast cancer risk in a Greek but not a Turkish population, is more frequent in Greeks (0.42) than in Turks (0.34), which may influence the association between the A blood group and disease status, although the O group frequency is similar between the two populations (0.40 and 0.43 in Greeks and Turks, respectively).

The variability of the minor allele frequency of SNP rs505922 also varies between populations: in a pilot study from the 1,000 Genomes Project [18], the frequency of rs505922 was 0.32 in YRI (Sub-Saharan Africa), 0.352 in CHB + JPT (China and Japan) and 0.417 in CEU (Caucasians from Utah). Although patients from ten European countries and Shanghai were included in the pancreatic GWAS study, the majority of patients were from a number of cohort studies from the United States. Similar results were achieved when all patients were included in the analysis and when only those of European ancestry were included [10]. In our study, all samples were collected from self-described white women living in Washington DC or rural Pennsylvania. Given the marked admixture in the United States, these 629 women likely represent a heterogeneous mixture of European ancestries. Thus, an association that may be detected in the more homogeneous Greek population may be masked by the genetic contributions from other European cultures represented in our American patient population.

## Conclusions

The minor allele frequency of SNP rs505922, which is in linkage disequilibrium with the non-O ABO blood types, did not differ between Caucasian American women with breast cancer and published HapMap frequencies. Further stratification into different tumor phenotypes also failed to reveal an association between rs505922 and any tumor characteristics. Together, these data suggest that unlike pancreatic cancer, where the minor rs505922 allele was associated with increased risk, especially of exocrine tumors, rs505922 and the resulting non-O blood types, are not associated with increased risk or less favorable tumor characteristics or prognosis in breast cancer.

## Competing interests

The author(s) declare that they have no competing interests.

## Authors’ contributions

SR generated the SNP data and contributed to writing the manuscript, CDS oversaw the sample collection and REE conceived of the study design, performed the statistical analysis and drafted the manuscript. All authors read and approved the final manuscript.

## Pre-publication history

The pre-publication history for this paper can be accessed here:

http://www.biomedcentral.com/1471-2350/13/41/prepub
